# Prevalence, patterns, and correlates of physical activity in Nepal: findings from a nationally representative study using the Global Physical Activity Questionnaire (GPAQ)

**DOI:** 10.1186/s12889-019-7215-1

**Published:** 2019-07-03

**Authors:** Zeljko Pedisic, Nipun Shrestha, Paul D. Loprinzi, Suresh Mehata, Shiva Raj Mishra

**Affiliations:** 1 Institute for Health and Sport, Victoria University, Melbourne, Australia; 2 Physical Activity Epidemiology Laboratory, Exercise Psychology Laboratory, Department of Health, Exercise Science and Recreation Management, The University of Mississippi, Oxford, USA; 3 Ministry of Health and Population, Kathmandu, Nepal; 4 Nepal Development Society, Chitwan, Nepal

**Keywords:** Physical activity, STEPS survey, Nepal

## Abstract

**Background:**

The promotion of a physically active lifestyle might help address the increasing burden of non-communicable diseases in Nepal. However, there is a lack of nationally representative estimates of physical activity (PA) prevalence in Nepal. The aim of this nationwide cross-sectional study was to determine domain-specific PA levels and the association of socio-demographic and lifestyle characteristics with total PA among Nepalese adults aged 15–69 years.

**Methods:**

The data were collected using self-administered questionnaires in a nationally representative sample of 4143 adults (66.5% females), comprised of both rural and urban populations in Nepal. PA levels were assessed using the Global Physical Activity Questionnaire (GPAQ).

**Results:**

Based on self-reported estimates, around 97% (95% confidence interval [CI]: 96–98%) of men and 98% (95% CI: 98–99%) of women were found to meet the recommended levels of PA. Both men and women reported high occupational PA, whilst most participants of both sexes did not report engaging in any leisure-time PA. A multiple regression analysis showed that less self-reported total PA was associated with older age, higher level of education, urban place of residence, never been married, being underweight, and smoking in both sexes and with overweight and obesity in males (*p* < 0.05 for all).

**Conclusion:**

According to self-reported estimates, majority of Nepalese men and women are meeting the recommended levels of PA. The total self-reported PA in Nepalese adults is high, because many of them have labour intensive jobs. Although older age, higher level of education, urban place of residence, never been married, being underweight, and smoking in both sexes, as well as overweight and obesity in males were inversely associated with self-reported PA, the overall level of PA in all these groups was very high. Given the high overall self-reported PA found in the current study, promoting more PA in Nepal may not be as important as in some other countries; not even in the population groups for which we found a negative association with PA. Nevertheless, future studies should examine whether a more balanced distribution of occupational and leisure-time PA would promote better health among Nepalese adults.

**Electronic supplementary material:**

The online version of this article (10.1186/s12889-019-7215-1) contains supplementary material, which is available to authorized users.

## Background

Physical inactivity has increased in epidemic proportions all around the globe over the past few decades [1]. It was estimated that around 30% of adults worldwide do not have a sufficient level of physical activity [2]. The large and increasing prevalence of physical inactivity is one of the major reasons for escalating trends of several non-communicable diseases (NCDs). Among various contributing factors to NCD-related mortality, physical inactivity alone is estimated to be responsible for 6–10% of deaths related to coronary heart disease, diabetes, and site-specific cancer [3]. Estimates for 2012 showed that almost three quarters of all NCD-related deaths globally occurred in low- and middle-income countries [4]. The General Assembly of the United Nations adopted a political declaration on the prevention and control of NCDs [5]. The declaration recognised the importance of strengthening national capacities to tackle NCDs, particularly in low- and middle-income countries. It also recognised the important role of the international community, including academic and research institutes, in assisting low- and middle-income countries in their efforts to effectively respond to the problem of NCDs.

Nepal is among the largest low-income countries according to population size. With urbanization, Nepal has undergone a rapid epidemiological transition, with NCDs such as cardiovascular disease, diabetes, and cancer topping the list. The promotion of a physically active lifestyle might help address the increasing burden of NCDs in Nepal [6–8]. However, maintaining individual physical activity levels may be challenging due to unavailability of parks, walking trails, and cycling lanes, especially in urban areas [9]. In 2003, a World Health Organization (WHO) report, based on the data collected among a sample of > 2000 adults in Kathmandu, the capital city of Nepal, found that 75% of men and 91% of women were inactive [10]. A much lower prevalence of physical inactivity (43.3%; 95% CI: 39.4–47.1%) was reported in a later study conducted in the outskirts of Kathmandu [11]. The prevalence estimates of physical inactivity in the general adult population vary significantly across world regions; from around 17% in Southeast Asia to around 43% in the eastern Mediterranean and Americas [2]. The variation is even larger between individual countries, with national prevalence estimates of physical inactivity ranging from less than 5% to more than 70% [2]. Population estimates of physical activity for specific countries are needed to inform national public health efforts [12, 13]. Only a few low-income countries have developed policies on physical activity [14, 15], possibly because of limited national epidemiological data needed to inform policy makers about their public health importance. There are limited and inconsistent data on population-level prevalence of physical activity in Nepal.

According to the social-ecological model, interventions that aim to increase physical activity should focus on intrapersonal, interpersonal, environmental, and policy-related correlates of this behaviour [16, 17]. Generic, non-country specific evidence shows that individual-level factors, such as older age, female sex, lower motivation for physical activity, lower self-efficacy, poorer health status, and less previous experience in physical activity are associated with lower levels of physical activity [18]. However, to design evidence-based interventions to promote physical activity in a specific country, it is necessary to understand the correlates of physical activity in that particular population. Population-based studies exploring correlates of physical activity among Nepalese adults are scarce.

Prevalence of physical activity in a population may change over time [19]. Some countries, such as Australia [20], Finland [21], and the US [22], therefore periodically update their national evidence on population-level physical activity. Updated knowledge on the distribution and determinants of physical activity will be helpful for policy makers to identify strategies for physical activity promotion in Nepal. Therefore, the aim of this study was to determine the prevalence, levels, patterns, and correlates of physical activity in a nationally representative sample of Nepalese adults.

## Methods

### Study design and participants

We used the “Non Communicable Diseases Risk Factors: STEPS Survey Nepal 2013” data. A detailed description of the survey methodology has been presented elsewhere [23]. In summary, a cross sectional, population-representative survey was conducted from January to June 2013. Sampling was done using the Probability Proportional to Size (PPS) method, ensuring proportionate representation of Nepal’s three ecological zones (mountain, hill, and Terai/plains). A sample of 4200 participants aged 15–69 was obtained (response rate = 89.8%). The participants were interviewed in their households by trained interviewers. Overall, 4113 participants (67.8% females) with valid data on physical activity were included in the analyses.

### Measures

Physical activity was assessed using the Global Physical Activity Questionnaire (GPAQ). The questionnaire asks about physical activity in the domains of work, transport**,** and leisure-time in a typical week. It also includes a question on the total time in a typical week spent in sedentary behaviour, that is, sitting or reclining while awake with a very low energy expenditure [24]. We presented the estimates of total and domain-specific physical activity in Metabolic Equivalent (MET)-minutes/week, that is, units of relative energy expenditure. Total self-reported physical activity was calculated as the sum across all three domains. The reliability of GPAQ found in an international study conducted in nine countries was moderate to high [25]. The validity of GPAQ tested against accelerometer and pedometer estimates ranged from 0.06 to 0.35, where for all countries except for Bangladesh it was above 0.23 [25].

Additionally, the following socio-demographic, and lifestyle characteristics were assessed in the survey: age in years (categorised as 15–29, 30–44, and 45–69 years), sex, education (no formal schooling, primary level, secondary level, and higher level), marital status (never married, currently married, and divorced/widowed/separated), smoking status (non-smoker and smoker), and place of residence (urban and rural). Body height was assessed using a portable stature scale, whilst body mass was measured by a digital weighing scale. Body Mass Index (BMI) was calculated as the ratio between body mass (kg) and squared body height (m^2^), and was further collapsed into the following categories: underweight (< 18.5 kg/m^2^), normal weight (18.5–24.9 kg/m^2^), overweight (25–29.9 kg/m^2^), and obesity (≥ 30 kg/m^2^).

### Data analysis

The distributions of self-reported physical activity and sedentary behaviour variables were positively skewed. Therefore, we presented these data using medians and their 95% confidence intervals. From the participants’ self-reported data, we also calculated the percentage of the sample meeting the physical activity recommendations for adults (≥150 min of moderate-intensity physical activity per week, or ≥ 75 min of vigorous-intensity physical activity per week, or an equivalent combination of the two intensities) and the recommendations for achieving additional health benefits (≥300 min of moderate-intensity physical activity per week, or ≥ 150 min of vigorous-intensity physical activity per week, or an equivalent combination of the two intensities), issued by the U.S. Department of Health and Human Services [26]. Furthermore, we used a multiple regression analysis to assess the relationship of socio-demographic and lifestyle characteristics (independent variables) with total self-reported physical activity levels (dependent variable). Non-normal distributions of residuals were detected in the regression models, and, therefore, we log-transformed the dependent variable. Sampling weights were used to obtain population-representative estimates. All analyses were performed using Statistical Package for Social Sciences (SPSS) version 16 (SPSS, Chicago, Illinois, USA), with the statistical significance threshold set at *p* < 0.05.

## Results

Socio-demographic and lifestyle characteristics of the participants in the current study are presented in Table 1. The sample included 68% women and 32% men. Most participants of both sexes were: from rural areas; currently married; of ‘normal’ BMI, and non-smokers. The most prevalent age category was 45–69 years in men and 30–44 years in women. In both sexes, the least prevalent education category was higher education. More women than men in the sample had no formal schooling.

**Table 1:** Sample characteristics

Variable/category	Males	Female
	n (%)	n (%)
Age (years)		
15–29	289 (22)	683 (24)
30–44	417 (31)	1141 (41)
45–69	630 (47)	983 (35)
Place of residence		
Urban	269 (20)	508 (18)
Rural	1067 (80)	2299 (82)
Marital status		
Never married	165 (12)	171 (6)
Currently married	1118 (84)	2452 (87)
Divorced/widowed/separated	53 (4)	184 (7)
Education level		
No formal schooling	299 (22)	1552 (55)
Primary	402 (30)	619 (22)
Secondary	532 (40)	564 (20)
Higher	103 (8)	72 (3)
Body mass index^a^		
Underweight	134 (10)	341 (12)
Normal weight	843 (65)	1727 (63)
Overweight	277 (21)	532 (19)
Obese	44 (3)	162 (6)
Smoking status^b^		
Smoker	410 (31)	355 (13)
Non-smoker	926 (69)	2452 (87)

^a^Calculated based on the self-reported height and weight

^b^Based on the single question “Do you currently smoke any tobacco products, such as cigarettes, cigars, pipes, bidis, hukahs or tamakhus?”

The median total self-reported physical activity in the population of Nepalese men and women was found to be 8400 and 7140 MET-minutes/week, respectively (Table 2). The highest self-reported energy expenditure was found in the work domain in both men and women. Most male and female participants did not report engaging in any leisure-time physical activity; hence the median energy expenditure in this domain was zero for both sexes. Both men and women reported engaging in sedentary behaviour on average 2 h a day. Self-reported physical activity levels by age×sex groups are presented in Additional file 1. Based on self-reported estimates, a vast majority of Nepalese men and women met physical activity recommendations [26] (Table 3).

**Table 2:** Sedentary behaviour and total, domain-specific, and intensity-specific physical activity levels of Nepalese adults aged 15–69 years

Physical activity	Median; Interquartile range (95% CI)^a^		*p*-value^b^
	Males	Females	
Domains			
Work^c^	5040; 8880 (4390–5040)	5040; 5760 (5040–5280)	0.943
Transport^c^	1680; 2520 (1680–1680)	1680; 1960 (1680–1680)	< 0.001
Leisure time^c^	0; 0 (0–0)	0; 0 (0–0)	< 0.001
Intensity bands			
Moderate intensity^d^	1155; 1142 (1124–1200)	1365; 1140 (1260–1440)	< 0.001
Vigorous intensity^d^	180; 840 (180–180)	0; 420 (0–0)	< 0.001
Total physical activity^c^	8400; 9240 (7920–8400)	7140; 7174 (6720–7560)	< 0.001
Total sedentary behaviour^e^	2; 2 (2–2.25)	2; 2 (2–2.25)	0.690

^a^95% confidence interval for median

^b^*p*-value of the independent samples median test

^c^MET-minutes/week

^d^Minutes/week

^e^Hours/day

**Table 3:** Prevalence of physical activity among Nepalese adults aged 15–69 years

Meeting PA recommendations	Percentage (95% CI)^a^		*p*-value^b^
	Males	Females	
Standard^c^	97 (96–98)	98 (98–99)	0.009
For additional health benefits^d^	93 (92–94)	96 (95–97)	< 0.001

^a^95% confidence interval for percentage

^b^*p*-value calculated using the two-proportion z-test

^c^≥ 150 min of moderate-intensity physical activity per week, or ≥ 75 min of vigorous-intensity physical activity per week, or an equivalent combination of the two intensities

^d^≥ 300 min of moderate-intensity physical activity per week, or ≥ 150 min of vigorous-intensity physical activity per week, or an equivalent combination of the two intensities

In the multivariate models, being 45–69 years old (vs. being 15–29 years old), never been married (vs. currently being married), having secondary education and higher education (compared to no formal schooling), living in an urban area (vs. living in a rural area), being a smoker (vs. not smoking), and being underweight (vs. ‘normal’ BMI) was associated with lower total self-reported physical activity (*p* < 0.05 for all) in both sexes (Table 4). Specifically, being 45–69 years old was associated with 20% (95% CI: 13–18%) and 12% (95% CI: 9–18%) lower total self-reported physical activity among men and women, respectively, when compared to the youngest age group. Being currently married was associated with 7% higher self-reported physical activity in both sexes (95% CI: 0–14% for males; 3–12% for females). Having secondary education was associated with 14% (95% CI: 6–20%) lower total self-reported physical activity in males and 6% (95% CI: 2–10%) lower self-reported physical activity in females. Having higher education was associated with 30% (95% CI: 19–43%) lower total self-reported physical activity in males and 10% (95% CI: 2–18%) lower self-reported physical activity in females. Living in an urban area was associated with 19% (95% CI: 12–25%) and 10% (95% CI: 5–12%) lower total self-reported physical activity among men and women, respectively. Smoking was associated with 11% lower self-reported physical activity in both sexes (95% CI: 5–16% for males; 6–16% for females). Being overweight and being obese was associated with 9% (95% CI: 2–14%) and 27% (95% CI: 11–43%), respectively, lower total self-reported physical activity in men, whilst we did not find such associations in women (*p* = 0.880 for overweight and *p* = 0.161 for obesity). Overall, the independent variables in the regression model explained in total 14 and 25% of variance of total self-reported physical activity in men and women, respectively.

**Table 4:** Results of multiple regression analyses with socio-demographic and lifestyle characteristics as independent variables and total physical activity level as the dependent variable

	Total physical activity^a^/B^b^ (95% CI)^c^			
	Males	*p*-value^d^	Females	*p*-value^d^
Age in years				
15–29	Ref		Ref	
30–44	-2 (− 8–4)	0.516	−1 (− 4–3)	0.702
45–69	− 20 (− 28 – − 13)	< 0.001	− 12 (− 18 – − 9)	< 0.001
Marital status				
Never married	Ref		Ref	
Currently married	7 (0–14)	0.032	7 (3–12)	0.001
Divorced/widowed/separated	11 (− 5–3)	0.194	−4 (− 11–4)	0.354
Education level				
No formal schooling	Ref		Ref	
Primary	−5 (−12–2)	0.124	4 (0–7)	0.028
Secondary	−14 (− 20 – − 6)	< 0.001	-6 (− 10 – − 2)	0.002
Higher	−30 (− 43 – −19)	< 0.001	− 10 (− 18 – − 2)	0.011
Place of residence				
Rural	Ref		Ref	
Urban	−19 (−25 – − 12)	< 0.001	−10 (− 12 – − 5)	< 0.001
Smoking status				
Non-smoker	Ref		Ref	
Smoker	− 11 (− 16 – − 5)	< 0.001	− 11 (− 16 – − 6)	< 0.001
Body mass index				
Underweight	−11 (− 18 – − 3)	0.006	−8 (− 11 – − 4)	< 0.001
Normal weight	Ref		Ref	
Overweight	−9 (− 14 – −2)	0.010	0 (− 4–3)	0.880
Obese	−27 (− 43 – − 11)	< 0.001	− 4 (− 11–2)	0.161

^a^Dependent variable was log-transformed prior to the analysis

^b^Percent difference (from the reference category) in the dependent variable associated with being in the given category (calculated based on the antilog of unstandardized regression coefficient)

^c^95% confidence interval for the unstandardized regression coefficient

^d^*p*-value for the unstandardized regression coefficient

## Discussion

This analysis of data from a nationally representative survey provides an insight into current levels and correlates of physical activity among Nepalese adults. We found a very high prevalence of sufficient physical activity in this population, which is much higher than in most other countries [2]. It may be that this finding was partially due to the fact that Nepal is predominantly an agrarian country, with a large percentage of people in labour-intensive jobs [27]. According to the national living standard survey [27], 64% of the population are in the agriculture sector, which explains the high level of occupational physical activity. Additionally, around 80% of the Nepalese population lives in rural areas, where, due to relatively poor access to transportation infrastructure for motor vehicles, walking is a common mode of daily commuting [28]. Furthermore, the high prevalence estimate might also be partially due to possible methodological issues. For example, over-reporting of physical activity levels is a common methodological problem in physical activity studies based on self-reports [29]. The validity of GPAQ estimates, when tested against total pedometer counts, was previously found to be poor in Bangladesh [25]; a low-income country in close proximity to Nepal. It might be that validity of GPAQ estimates in Nepal is also poor. This should be examined in future validation studies for GPAQ conducted in the Nepalese context. Besides, the translation and cultural adaptation of GPAQ for the purpose of surveys in Nepal, may have affected the comparability with studies from other countries. Finally, individual responses to questions about physical activity may be affected by cultural factors [29], which may have also been the case in this study.

Furthermore, in the current study, the prevalence of self-reported physical activity was found to be much higher than in two previous Nepalese studies [10, 11]. It should be noted, however, that these studies were not conducted in nationally-representative samples, which likely reduced their comparability with the current study. One was conducted in a sample of adults from five out of overall 35 wards in Kathmandu metropolitan area [10], whilst the other one only included participants from the outskirts of Kathmandu [11]. Our regression analyses showed that living in an urban area is negatively associated with self-reported physical activity level. The place of residence of participants in both previous studies was in or around the largest Nepalese city, whilst a large proportion of participants in the current study were from other, mostly rural, areas of Nepal. The association between rural/urban place of residence and physical activity level may, therefore, partially explain the higher prevalence of self-reported physical activity found in the current study.

The high level of self-reported occupational physical activity in Nepalese adults that we found may be explained by the fact that many jobs in Nepal are still labour-intensive. Our finding of high self-reported occupational physical activity among Nepalese adults concurs with results from a previous study in Nepal [11], from other studies in Asia [19, 30], and internationally [13]. Previous studies found that people who are highly active at work are more likely to be inactive in leisure time [31, 32]. This might explain our findings suggesting that most Nepalese adults do not engage in any physical activity in leisure time, whilst engaging in very high amounts of occupational physical activity. Furthermore, our findings can be considered from three perspectives: [i] a traditional paradigm that deems physical activity in all domains equally healthy, as reflected in some physical activity guidelines [33, 34]; [ii] an emerging paradigm which suggests that physical activity in the work domain may not be as beneficial for health as leisure-time physical activity [35–38] and that a high volume of occupational physical activity may even be detrimental to health [39–41]; and [iii] the emerging time-use epidemiology paradigm suggesting that an optimal balance between the time spent in different components of time use (e.g., leisure-time physical activity, occupational physical activity, household physical activity, transport-related physical activity, sedentary behaviour, and sleep) is needed for good health [42]. If we were to interpret our findings within the first paradigm, we could conclude that no physical activity interventions are needed among Nepalese adults, because their total physical activity is already very high. However, if we consider our findings within the second or third paradigm, it might be that measures to achieve a more balanced distribution of occupational and leisure-time physical activity are needed to promote better health among Nepalese adults. Further evidence from epidemiological studies among Nepalese adults is needed to make concrete recommendations on this matter.

We found that lower level of self-reported physical activity was associated with older age, higher level of education, urban place of residence, never been married, being underweight, and smoking in both sexes and with overweight and obesity in males. However, it is important to note that the overall level of self-reported physical activity in all these groups was very high, despite the significant inverse associations we observed.

Nepalese adults aged 45 and above were less likely to be active than their younger counterparts; which is consistent with findings from previous studies [43]. Furthermore, having a higher education qualification was associated with lower levels of self-reported physical activity. This is also in accordance with the findings of previous studies in different countries [13, 44, 45]. Educated people in Nepal are more likely to have sedentary jobs, which is likely the reason for their lower total self-reported physical activity levels. Furthermore, the unprecedented transition from rural to urban areas that is currently happening in Nepal is adding fuel to ever increasing burden of NCDs. The government has failed to create adequate infrastructures in urban areas like parks, walking and running trails that facilitate greater physical activity. Moreover the roads are generally not safe for pedestrians [46, 47], largely due to non-existent pavement or cycling lanes, encroachment of pavements by street vendors, potholes, traffic congestion, absence of strict laws for traffic rules offenders, and air pollution from dust and vehicular emissions. These factors have made accumulation of leisure-time physical activity in cities almost impossible. The urban residents were found to have lower total self-reported physical activity than rural residents. Nevertheless, most of the urban residents were still found to be sufficiently active. It might be because of rising poverty in urban areas in Nepal [48], with increasing number of young people of very low socioeconomic status migrating to urban areas in search of better livelihood. These people are mostly employed in manual jobs requiring high levels of physical activity [49]. Furthermore, smokers reported less physical activity than their non-smoking counterparts. Smoking and physical inactivity may work synergistically, escalating the risk of chronic disease and premature death [50]. Hence, this subgroup of population may need special consideration in physical activity promotion. This is also important, because physical activity promotion among smokers has been shown to reduce the incidence of lung cancer [51]. It should be noted, however, that promoting smoking cessation should generally be the priority intervention in this population group. This seems particularly true in Nepal, given that the total self-reported physical activity level among smokers is already very high. Furthermore, we also found that self-reported physical activity was generally higher in those with a ‘normal’ BMI. However, given the fact that high self-reported physical activity was observed even among those categorised as obese, physical activity interventions on their own might, therefore, not be the strategy of choice when trying to tackle obesity in Nepal.

Less than one third of variance of self-reported physical activity was explained by the socio-demographic and lifestyle variables included in the regression model. Several other socio-demographic, health-related, psychological, interpersonal, and environmental correlates that were not assessed in this survey, might play important roles in shaping adults physical activity behaviour [18]. When possible, future studies of physical activity in Nepal should consider including more explanatory variables in their models.

This study was based on data from a large national sample consisting of both urban and rural populations in Nepal. However, as this was a cross-sectional study, causality cannot be inferred from our findings. Furthermore, we relied on self-reported physical activity data that might be subject to recall and social desirability biases [29]. Nevertheless, the use of standardised and validated questionnaires, such as GPAQ, has been recommended in physical activity surveillance, particularly in countries that cannot afford collecting data on population-level physical activity using motion sensors [52].

## Conclusion

Based on self-reported data, this study found that a vast majority of Nepalese men and women are meeting the recommended levels of physical activity. A lot of physical activity seems to be accumulated in the work domain due to labour intensive jobs, whilst most people do not report engaging in any leisure-time physical activity. Older age, higher level of education, urban place of residence, never been married, being underweight, and smoking in both sexes, as well as overweight and obesity in males, were inversely associated with self-reported physical activity. Despite that, the overall level of self-reported physical activity observed in all these groups was very high. Given the high overall self-reported physical activity found in the current study, promoting more physical activity in Nepal may not be as important as in some other countries; not even in the population groups for which we found a negative association with physical activity. Nevertheless, future studies should examine whether a more balanced distribution of occupational and leisure-time physical activity would promote better health among Nepalese adults.

## Additional file

Additional file 1: **Table S1.** Sedentary behaviour and total and domain-specific physical activity levels of Nepalese adults by age groups. (PDF 82 kb)

## Data Availability

The datasets used and/or analysed during the current study are available from the corresponding author on reasonable request.

## Abbreviations

BMI: Body Mass Index
GPAQ: Global Physical Activity Questionnaire
NCDs: Non-communicable diseases
PA: Physical activity
PPS: Probability Proportional to Size
SPSS: Statistical Package for Social Sciences
WHO: World Health Organization
