# Supplementary material for: Prevalence, patterns, and correlates of physical activity in Nepal: findings from a nationally representative study using the Global Physical Activity Questionnaire (GPAQ)
Source: BMC Public Health. 2019 Jul 3;19:864. doi: 10.1186/s12889-019-7215-1 (PMC6610855; doi:10.1186/s12889-019-7215-1)
Supplement: Supplementary file 1 — Table S1. Sedentary behaviour and total and domain-specific physical activity levels of Nepalese adults by age groups. (PDF 82 kb) [file 12889_2019_7215_MOESM1_ESM.pdf]

Additional table 1. Sedentary behaviour and total and domain-specific physical activity levels of Nepalese adults by age groups

Physical activity	Median; Interquartile range (95% CI)[a]					
	Males			Females		
	15 – 29 years	30 – 44 years	45 – 69 years	15 – 29 years	30 – 44 years	45 – 69 years
**Domains**						
Work[b]	4632; 8520 (4200 – 5040)	6720; 11610 (5880 – 7560)	3840; 7560 (3360 – 4200)	5040; 5592 (5040 – 5040)	6035; 6468 (5040 – 6720)	4200; 5830 (4200 – 4200)
Transport[b]	1680; 2520 (1680 – 1680)	1680; 2400 (1680 – 1680)	1680; 2520 (1680 – 1680)	1680; 2100 (1680 – 1680)	1680; 1680 (1680 – 1680)	1680; 1800 (1680 – 1680)
Leisure time[b]	0; 1774 (0 – 0)	0; 0 (0 – 0)	0; 0 (0 – 0)	0; 0 (0 – 0)	0; 0 (0 – 0)	0; 0 (0 – 0)
**Intensity bands**						
Moderate intensity[c]	1155; 1254 (1050 – 1230)	1260; 1050 (1155 – 1290)	1050; 1050 (1050 – 1050)	1470; 1094 (1470 – 1470)	1440; 1080 (1260 – 1470)	1260; 1260 (1140 – 1260)
Vigorous intensity[c]	280; 859 (240 – 315)	240; 1038 (180 – 360)	0; 630 (0 – 0)	0; 366 (0 – 0)	0; 591 (0 – 98)	0; 180 (0 – 0)
Total physical activity[b]	8520; 9120 (8400 – 8760)	9360; 10920 (9240 – 9720)	6720; 8400 (6660 – 6720)	7420; 7018 (7413 – 7560)	8040; 7253 (7360 – 8400)	5880; 6720 (5760 – 6000)
Total sedentary behaviour[d]	2; 2 (2 – 2)	2; 1.67 (2 – 2)	2; 2 (2 – 2)	3; 2 (2 – 3)	2; 1 (2 – 2)	2; 2 (2 – 2)

[a]95% confidence interval for median

[b]MET-minutes/week

[c]Minutes/week

[d]Hours/day
